# Selective removal of lead ions from aqueous solutions using 1,8-dihydroxyanthraquinone (DHAQ) functionalized graphene oxide; isotherm, kinetic and thermodynamic studies†

**DOI:** 10.1039/c7ra13603j

**Published:** 2018-02-02

**Authors:** Mohammad Khazaei, Simin Nasseri, Mohammad Reza Ganjali, Mehdi Khoobi, Ramin Nabizadeh, Elham Gholibegloo, Shahrokh Nazmara

**Affiliations:** Department of Environmental Health Engineering, School of Public Health and Research Center for Health Sciences, Hamadan University of Medical Sciences Hamadan Iran; Department of Environmental Health Engineering, School of Public Health, Tehran University of Medical Sciences P. O. Box: 14155-6446 Tehran Iran naserise@tums.ac.ir +98 2188950188 +98 2188954914; Center for Water Quality Research, Institute for Environmental Research, Tehran University of Medical Sciences Tehran Iran; Center of Excellence in Electrochemistry, Faculty of Chemistry, University of Tehran Tehran Iran; Biosensor Research Center, Endocrinology & Metabolism Molecular-Cellular Sciences Institute, Tehran University of Medical Sciences Tehran Iran; Department of Pharmaceutical Biomaterials and Medical Biomaterials Research Center, Faculty of Pharmacy, Tehran University of Medical Sciences Tehran Iran; Department of Chemistry, Faculty of Science, University of Zanjan Zanjan Iran

## Abstract

An anthraquinone – graphene structure was fabricated and applied for the removal of lead(ii) from aqueous solution. The equilibrium occurred in about 10 min revealing the high adsorption rate at the beginning of the process. The maximum Pb(ii) adsorption capacity of the Fe_3_O_4_@DHAQ_GO nanocomposite was about 283.5 mg g^−1^ that was observed at 323 K and pH 5.5. The Pb(ii) adsorption ability increased with the increasing pH. The isotherm and kinetic studies indicated that the Sips isotherm model and the linear form of the pseudo-second kinetic model had a better fit with the experimental results. The positive value of Δ*H*^0^ indicated endothermic interactions between Pb(ii) and Fe_3_O_4_@DHAQ_GO. The negative Δ*G*^0^ indicated that the reactions are spontaneous with a high affinity for Pb(ii). The positive Δ*S*^0^ values indicated increasing randomness at the solid–solute interface during the adsorption process. The selective removal of Pb(ii) by the nanocomposite confirms the presence of higher-affinity binding sites for Pb(ii) than Cd(ii), Co(ii), Zn(ii), and Ni(ii) ions. Furthermore, the Fe_3_O_4_@DHAQ_GO nanocomposite revealed an excellent preferential adsorbent for Pb(ii) spiked in drinking water samples containing natural ion matrices. EDTA-2NA 0.01 N was found to be a better elution agent than HCl 0.1 M for the nanocomposite regeneration. After five adsorption/desorption cycles using EDTA-2NA 0.01 N, more than 84% of the adsorbed Pb(ii) was still desorbed in 30 min. Capturing sub-ppm initial concentrations of Pb(ii) and the capability to selectively remove lead from drinking water samples make the Fe_3_O_4_@DHAQ_GO nanocomposite practically convenient for water treatment purposes. High adsorption capacity and facile chemical synthesis route are the other advancements.

## Abstract 2

The Fe_3_O_4_@DHAQ_GO nanocomposite can serve as an efficient adsorbent for the selective removal of lead from polluted water.

## Graphical abstract

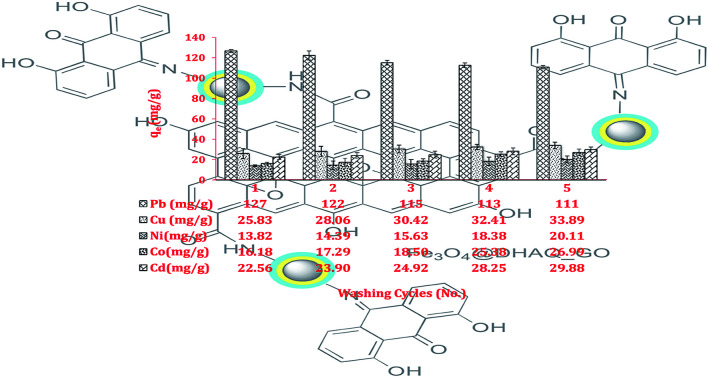

## 1. Introduction

Lead ions are a severe environmental concern and can contaminate drinking water resources.^1,2^ The maximum contaminant level (MCL) of Pb^2+^ for drinking water is 10 ppb set by EPA and national standard organizations.^3,4^ The strict limitations on discharge effluents containing Pb^2+^ into natural water bodies are due to the high toxicity potential for vital organs such as brain and kidney.^2^

Different methods are currently applied for the removal of high concentrations of lead ion that can be found in industrial wastewaters;^5–8^ whereas only a few methods *e.g.* using functionalized adsorbents^9,10^ and membrane technologies^11^ can be adapted for the capturing of low concentrations (around 1 ppm) commonly occurring in drinking water sources. Furthermore, avoiding alteration of the natural ion matrices of drinking waters during the removal of a certain target contaminant is a consideration especially for membrane-based water treatment technologies.^12,13^ New generation adsorbents such as graphene oxide and carbon nanotubes show metal adsorption capacities much more than those of traditional adsorbents.^14^ For example, the ordinary adsorption capacity of activated carbon is less than 70 mg g^−1^, whereas graphene oxide nanosheets are capable of reaching an adsorption capacity of 4000 mg g^−1^.^15^

Graphene oxide is an emerging carbon-based nanomaterial that has revealed the promising adsorptive properties. Despite the graphene (G) and reduced graphene oxide (RGO), the graphene oxide (GO) creates a highly stable aqueous dispersion.^16^ This property leads to increase the effective contacts with target contaminants without vigorous mechanical mixing. The dispersability properties of GO is attributed to the plenty hydrophilic functional groups covering the GO flakes.^17^ The GO flake surface contains various functional groups including epoxy and hydroxide, whereas the edge of flakes mainly contains the carboxylic groups.^18^

In recent years, using Pb^2+^ selective membrane electrodes (ISE) have been extensively studied with different active materials to determine lead ion concentration in water and wastewater.^19,20^ The active materials are mainly consisting of ligands or Schiff bases, which are known as ionophores.^21^ It has been revealed that some ionophores such as anthraquinone,^22–24^ methacrylate,^25^ and nucleic acids^26^ have the selective affinity to lead ion. The main drawback regarding to the most of ionophores is their hydrophobic nature which makes them unusable to create aqueous solution for the lead ion removal.^27^ Using GO flakes as the aqueous dispersion agents can provide an appropriate platform for the attachment of ionophores and producing a water dispersible GO-ionophore composite.

1,8-Dihydroxyanthraquinone (DHAQ), namely Dantron is a dye intermediate and a medicine.^27,28^ Furthermore, some works report the high affinity of DHAQ as a ligand to form stable complexes with Pb^2+^.^20,24^ In this study, DHAQ was used as an ionophore agent in the structure of Fe_3_O_4_@SiO_2_–GO to form the Fe_3_O_4_@DHAQ_GO nanocomposite and aimed to have Pb^2+^ selective removal property from aqueous solutions.

## 2. Materials and methods

### 2.1. Materials

Graphite powder (particle size 20 μm), tetraethyl orthosilicate (TEOS), (3-aminopropyl) triethoxysilane (APTES), *n*-hydroxysuccinimide (NHS), 1-ethyl-3-(3-dimethyl aminopropyl) carbodiimide (EDC·HCl), and 1,8-dihydroxyanthraquinone (DHAQ) were purchased from Sigma-Aldrich, Ltd. Co. All other chemicals such as sodium nitrate (NaNO_3_), potassium permanganate (KMnO_4_), sulfuric acid (H_2_SO_4_), hydrochloric acid (HCl), hydrogen peroxide aqueous solution (H_2_O_2_), iron chloride hexahydrate (FeCl_3_, 6H_2_O), and iron chloride tetrahydrate (FeCl_2_, 4H_2_O) were of reagent grade and used without further purification.

### 2.2. Preparation of Fe_3_O_4_@SiO_2__GO

Our previous work reported the fabrication of graphene oxide (GO), Fe_3_O_4_ magnetic nanoparticles, Fe_3_O_4_@SiO_2__NH_2_ nanoparticles, and Fe_3_O_4_@SiO_2__GO nanocomposite.^1^ The preparation of GO was based on using sulfuric acid as digestion agent, and H_2_O_2_ for the oxidation of graphite.^29^ Co-precipitation method was used to prepare Fe_3_O_4_ magnetic nanoparticles.^30^ Then, NH_2_-groups were applied as linkers to create covalent bonds between Fe_3_O_4_ magnetic nanoparticles and GO. Consequently, covering APTES and TEOS on the Fe_3_O_4_ magnetic nanoparticles produces Fe_3_O_4_@SiO_2__NH_2_.^31,32^ Finally, a condensation reaction between the carboxylic groups (COO–) of GO and the amine groups (NH_2_–) of Fe_3_O_4_@SiO_2_ was prepared for the fabrication of Fe_3_O_4_@SiO_2__GO nanocomposite.^32^

### 2.3. Preparation of Fe_3_O_4_@DHAQ_GO

**Fig. 1:**
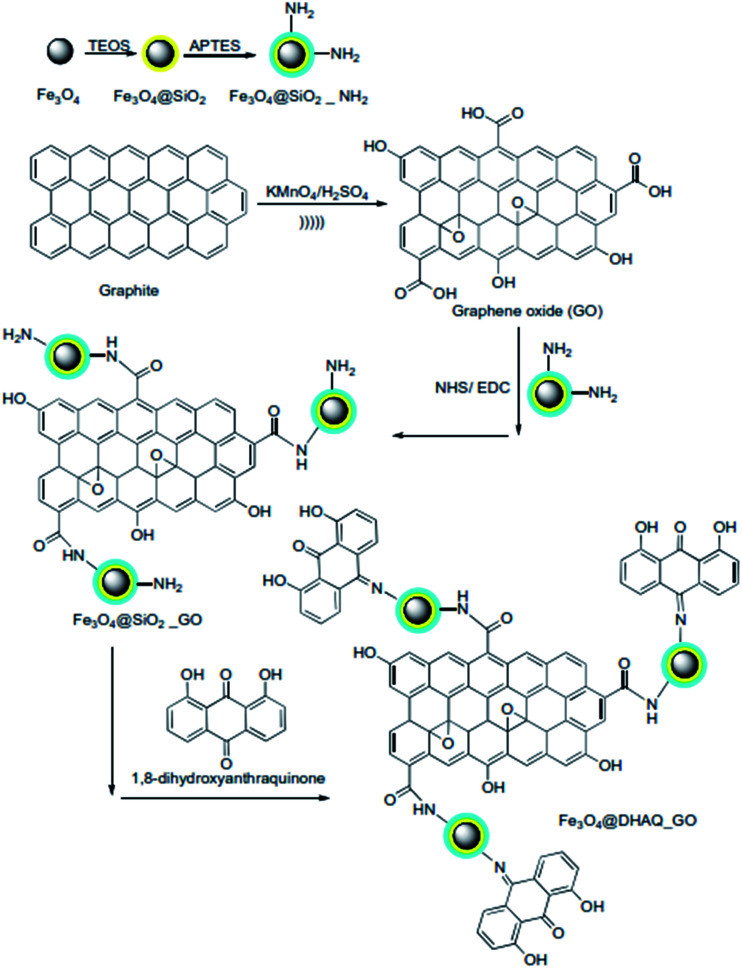
Schematic of the chemical path to synthesis Fe_3_O_4_@DHAQ_GO nanocomposite.

200 mg DHAQ was added into 50 mL DMF followed by mild stirring for 3 hours. Then, 200 mg EDS and 100 mg NHS were added and pH was adjusted between 4 to 6 followed by vigorous mixing for 2 hours at room temperature. After that, 0.5 g Fe_3_O_4_@SiO_2__GO was dispersed into the mixture and mixing was continued up to 12 hours. Finally, dispersed solid was separated *via* centrifuge (12 000 rpm, 10 min), washed with deionized water, and dried to obtain Fe_3_O_4_@DHAQ_GO. Schematic of the synthesis path applied for the fabrication of Fe_3_O_4_@DHAQ_GO nanocomposite was presented in Fig. 1. As revealed, 1,8-dihydroxyanthraquinone attaches to amine group linked with Fe_3_O_4_ nanoparticle.

### 2.4. Instrumentation

The prepared nanocomposite was characterized applying SEM (MIRA3, TESCAN®, Czech), AFM (SPM, VEECO®, USA), FTIR (Spectrum One, Perkin-Elmer®, USA), XRD (Philips®, Netherlands), UV-Visible spectrophotometer (Perkin-Elmer®, USA), TEM (EM900, Zeiss®, Germany), TGA (TGA 4000, Perkin-Elmer®, USA), and pHpzc. The initial and final concentration of Hg(ii) were measured by using an ICP-OES (ARCOS, SPECTRO®, Germany). pH was adjusted by using a MITEC-965 (micra®, India) pH meter. A thermostatic shaker (Innova 4340, Eppendorf, Germany) was used to study the batch experiments.

### 2.5. Characterization

A Hitachi-S4160 scanning microscope were used to provide SEM images (Tokyo, Japan). The AFM measurements were obtained by using a Nanoscope V multimode atomic force microscope (Veeco Instruments, USA). Samples prepared for the AFM measurements contained dispersions of GO/methanol solutions (70 mg mL^−1^) smeared on a fresh mica surface and allowed drying in the air.^33^

### 2.6. Adsorption experiments

A typical adsorption experiment was established by adding 10 mg Fe_3_O_4_@DHAQ_GO into a 100 mL solution containing Pb^2+^ ions at room temperature. Varied initial concentrations of Pb^2+^, from 1 mg L^−1^ to 10 mg L^−1^, were used and for all the Pb^2+^ aliquots, the pH value was kept on 7 applying buffer solutions. The mixing rate was constant at 150 rpm for the all solutions.

An external magnetic field was used for the removal of adsorbent after the adsorption time. The equilibrium adsorption capacity (*q*_e_, mg g^−1^) of Pb^2+^ was determined by the following equation.1
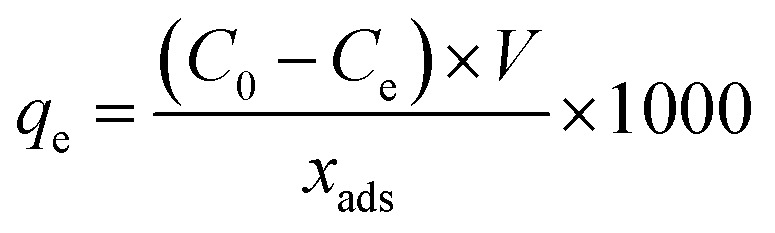
where, *C*_0_ and *C*_*t*_ are the Pb^2+^ initial and final concentrations (mg L^−1^), *x*_ads_ is the adsorbent mass (g), *V* is the reactor volume (L), and 1000 is a conversion factor.

A Spectro Arcos ICP-optical emission spectrometer (SPECTRO Analytical Instruments, Kleve, Germany) was used for the measurement of Pb^2+^ concentrations.

The parameters of isotherm and kinetic equations were determined by applying Solver engine of Microsoft Excel spreadsheet software^34^ based on nonlinear forms of the equations.

### 2.7. Selectivity study

Two independent studies were conducted to investigate the capability of Fe_3_O_4_@DHAQ_GO nanocomposite for the selective capturing of Pb^2+^ from water: binary ion study; including aliquots contained binary ion matrices (Pb^2+^/Cu^2+^, Pb^2+^/Cd^2+^, Pb^2+^/Zn^2+^, and Pb^2+^/Co^2+^) and selective removal of Pb^2+^ from natural water samples; including drinking water samples spiked with Pb^2+^ ions. The concentration of metal ions was measured by using ICP-OES. The distribution coefficient *K*_d_ (mL g^−1^), selectivity coefficient *k*, and the relative selectivity coefficient *k* were determined by eqn (2)–(4), respectively.2
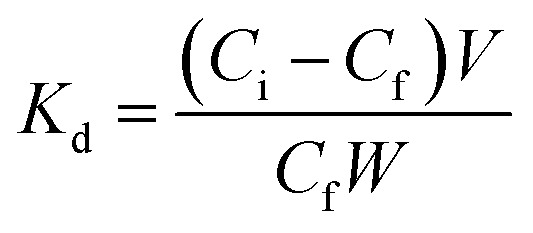
3
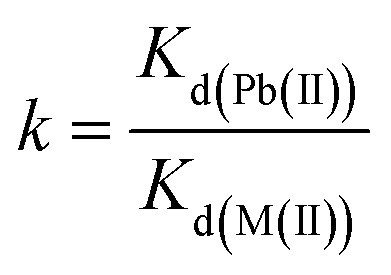
4
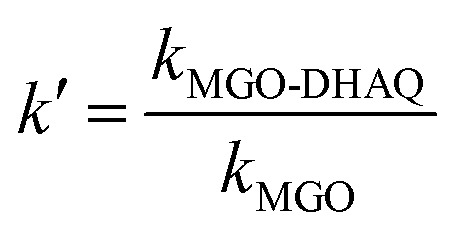
where, *C*_i_ and *C*_f_ are the initial and final concentrations of metal ions, respectively. *K*_d(Pb(II))_ and *K*_d(M(ii))_ are the distribution coefficient of Pb^2+^ and metal (M) ions, respectively. *k*_MGO-DHAQ_ and *k*_MGO_ are the selectivity coefficient of Fe_3_O_4_@DHAQ_GO and Fe_3_O_4_@SiO_2_–GO, respectively.

### 2.8. Desorption and regeneration

Pb^2+^ in solution (25 mL, 2.45 mg L^−1^) was adsorbed onto Fe_3_O_4_@DHAQ_GO (30 mg L^−1^) at pH 7 for 1 h and then the adsorbents were separated by applying an external magnetic field and the residual quantity of metal ions was determined by ICP-OES. After that, the adsorbents were regenerated in 25 mL Erlenmeyer flask containing 10 mL 0.02 mol L^−1^ eluent to completely leach metal ions at room temperature for 6 h. The concentration of metal ions released from adsorbent into the aqueous phase was measured by ICP-OES. Desorption ratio (*D*) was determined by using the following equation:5
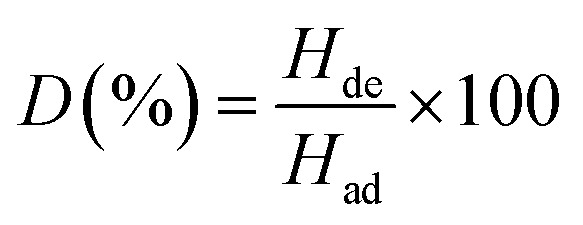
where, *H*_de_ (mg L^−1^) is the amount of metal ion desorbed into the elution medium. *H*_ad_ (mg L^−1^) is the amount of metal ion adsorbed onto the Fe_3_O_4_@DHAQ_GO nanocomposite.

## 3. Results and discussion

It is well known that various derivatives of anthraquinone are able to form stable complexes with a variety of metal ions in some non-aqueous solvents^35,36^ and anthraquinone–lead(ii) complexes are among the most stable ones.^37,38^ Applying the graphene oxide provides the active sites for the anthraquinone that can be covalently bonded and produced a hydrophilic property which is appropriate for the adsorption of Pb^2+^ in the aqueous solution.

### 3.1. Characterization studies

**Fig. 2:**
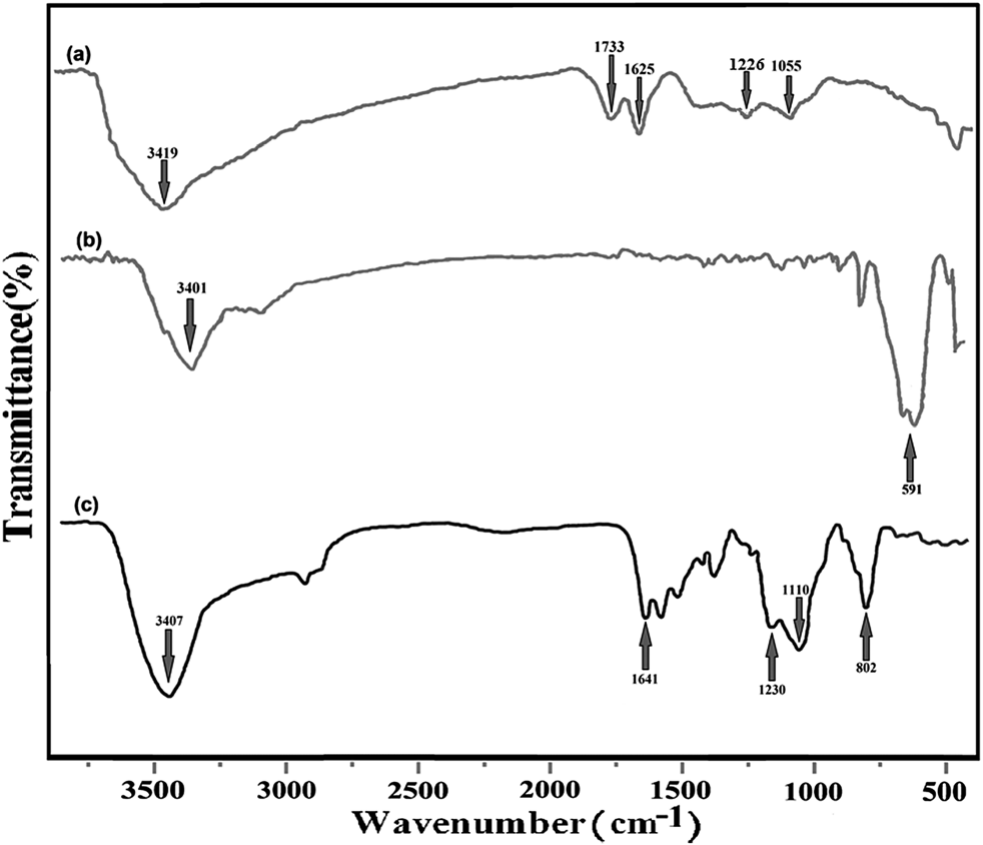
FT-IR spectra of GO (a), Fe_3_O_4_@SiO_2_–GO (b), and Fe_3_O_4_@DHAQ_GO (c).

The FT-IR spectra for GO, Fe_3_O_4_@SiO_2_–GO, and Fe_3_O_4_@DHAQ_GO are presented in Fig. 2. The stretchings C–O (1055 cm^−1^), C–OH (1226 cm^−1^), C–O carbonyl (1733 cm^−1^), and O–H hydroxide (3419 cm^−1^)^39–41^ can be observed in the FT-IR spectrum of GO (Fig. 2(a)). The stretching assigned to the adsorbed water molecules is observed at 1621 cm^−1^ assigning also to the skeletal vibrations of un-oxidized graphite.^42,43^

In Fig. 2(b), the spectrum of Fe_3_O_4_@SiO_2_–GO is depicted. It shows the vibration of Fe–O stretching at 591 cm^−1^ and an intense stretching around 3400 cm^−1^. Although, it can be attributed to the remaining water on the surfaces of Fe_3_O_4_ nanoparticles.^44^

Fig. 2(c) depicts the FT-IR spectrum of Fe_3_O_4_@DHAQ_GO. As shown, a vibration is observed at 3401 cm^−1^ assigning to the N–H stretching. Furthermore, the peak at 1733 cm^−1^, observed in Fig. 2(a), is disappeared and a new wide peak of C=N stretching is appeared at 1641 cm^−1^. The vibration of C–N stretching is appeared at 1230 cm^−1^.^45^ The obvious peaks at 802 and 1110 cm^−1^ can be attributed to the Si–O vibrations. The FTIR spectra confirmed that APTES functionalized Fe_3_O_4_ has been bonded covalently to GO nanosheets *via* the amide linkage.^46^

Fig. S1† depicts field emission SEM images of GO, Fe_3_O_4_@SiO_2_–GO, and Fe_3_O_4_@DHAQ_GO nanoparticles. From Fig. S1(a),† it can be observed that GO is partially transparent and 2- or 3-layered graphene oxides are formed.^47,48^ From Fig. S1(b),† the spherical Fe_3_O_4_@SiO_2_–NH_2_ nanoparticles having 50–60 nm diameters are identified, which finally have been enveloped by GO layers producing aggregated morphologies of Fe_3_O_4_@DHAQ_GO as shown in Fig. S1(c)†.

**Fig. 3:**
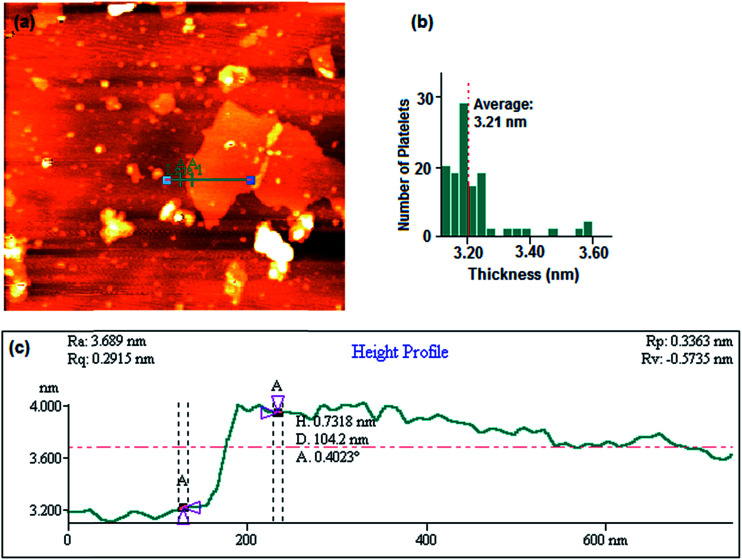
Tapered mode AFM topography scan. Exfoliated graphene oxide deposited on a freshly cleaved mica surface (a), histogram of platelet thicknesses from images of 138 platelets (the mean thickness is 3.21 nm) (b), height profile through the green line (Line 1) presented in (a). Cross-section A–A through the sheet shown in (a) exhibiting a height of 0.732 nm (c).

Fig. 3 illustrates the tapered mode AFM topography scan. A single platelet of GO laid on a freshly cleaved mica surface can be observed in Fig. 3(a) and (b) represents a frequency histogram of platelets thicknesses having the mean thickness of 3.21 nm. Height profile of the green line (Line 1 in Fig. 3(a)) presents a height of 0.732 nm in cross-section A–A as shown in Fig. 3(c).

Fig. S2† presents thermal gravimetric analysis (TGA) of Fe_3_O_4_ magnetic nanoparticles, Fe_3_O_4_@SiO_2_–GO, Fe_3_O_4_@DHAQ_GO, and graphene oxide. As revealed, major weight losses were occurred between 150 and 350 °C attributing to CO, CO_2_ released from labile functional groups.^48,49^ Slower rate of mass loss was detected between 350 and 650 °C assigning to the removal of some stable oxygenated functional groups. Similar trends of weight loss were observed in Fe_3_O_4_@SiO_2_–GO and Fe_3_O_4_@DHAQ_GO. The Fe_3_O_4_@DHAQ_GO weight loss was 13.5% more than those of Fe_3_O_4_@SiO_2_–GO in 740 °C attributing to the presence of 1,8-dihydroxyanthraquinone in the structure of Fe_3_O_4_@DHAQ_GO.^50^

Fig. S3† shows the XRD patterns of GO and Fe_3_O_4_@SiO_2_–GO. GO sharp diffraction peaks observable at 2*θ* = 12.24° and 42.83° are attributed to the reflections of (002) and (101), respectively. Furthermore, six typical peaks at about 2*θ* = 30.4, 35.6, 43.1, 54.1, 57.7 and 62.5° are observed for Fe_3_O_4_@SiO_2_–GO, attributing to indices (220), (311), (400), (422), (511) and (440), respectively. Appropriate match of intensities and positions of above mentioned diffraction peaks confirming by pure magnetite JCPDS card (75-1610).^51^ As represented in XRD patterns corresponding to Fe_3_O_4_@SiO_2_–GO, the reflection peak (002) belonging to GO was disappeared. It is assumed that the GO sheets cover the Fe_2_O_3_ nanoparticles and it hinders the stacking of sheets to create a crystalline arrangement.^52^

The vibration sample magnetization (VSM) was used to determine the magnetic characteristics of fabricated materials contained Fe_3_O_4_. Fig. S4† shows that the maximum saturation magnetizations of Fe_3_O_4_ NPs, Fe_3_O_4_-APTES, GO@SiO_2_–Fe_3_O_4_, and Fe_3_O_4_@DHAQ_GO were 53.2, 40.1, 19.7, and 13.5 emu g^−1^, respectively. Decreasing the maximum saturation magnetizations can be ascribed to the Fe_3_O_4_ nanoparticles covering consecutively by APTES, SiO_2_–GO, and DHAQ.

**Fig. 4:**
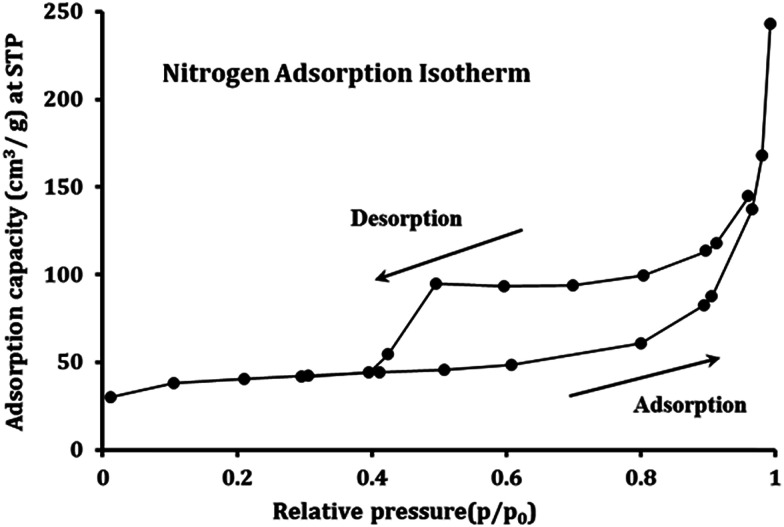
Nitrogen adsorption–desorption isotherms for Fe_3_O_4_@DHAQ_GO nanocomposite.

Fig. 4 presents the nitrogen adsorption isotherm of Fe_3_O_4_@DHAQ_GO nanocomposite. The surface area of 215 m^2^ g^−1^ was obtained that is relatively lowered than those reported for pristine GO.^53^ It seems that the agglomeration of Fe_3_O_4_ NPs and GO nanosheets tend to an shrinking effect on the GO nanosheets causing the decrease of free surface area^48^ as observed in Fig. S1.† The average pore size of Fe_3_O_4_@DHAQ_GO was determined to be about 9.26 nm identifying the mesopore structure of the adsorbent.

### 3.2. Adsorption experiments

#### 3.2.1. Adsorption isotherm

The isotherm models Langmuir (eqn (6)), Freundlich (eqn (7)), and Sips (eqn (8)) were applied to investigate the effect of equilibrium concentrations of Pb^2+^ (*C*_e_) on the equilibrium adsorption capacities (*q*_e_) of Fe_3_O_4_@DHAQ_GO nanocomposite.6
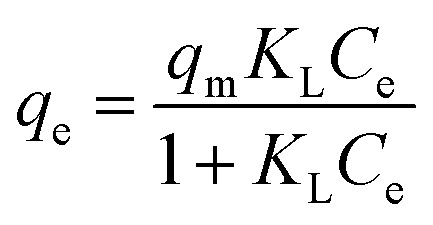

7 *q*_e_ = *K*_f_*C*^1/*n*^_e_^_F_^

8
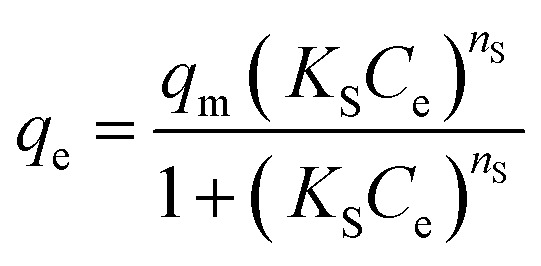
where, *K*_L_ is the Langmuir adsorption constant (L mg^−1^) and *q*_m_ represents the maximum adsorption capacity attributing to the complete monolayer coverage of the adsorbent (mg g^−1^). Furthermore, *K*_F_ (mg g^−1^) and *n*_F_ (unit less) are the Freundlich constants. *K*_S_ (L g^−1^) and *n*_S_ are the Sips equation parameters denoting the affinity constant and surface heterogeneity, respectively.^54,55^

**Fig. 5:**
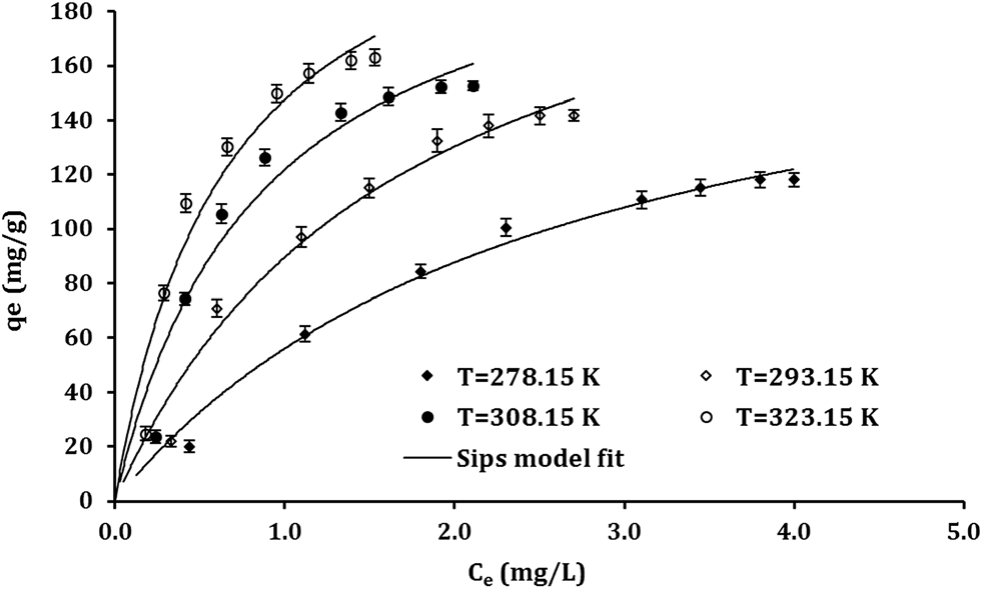
Adsorption isotherms of Pb^2+^ on Fe_3_O_4_@DHAQ_GO nanocomposite at different temperatures. (Adsorbent dosage 100 mg L^−1^; volume of solution 100 mL; pH 7; Pb^2+^ initial concentration range 1–10 mg L^−1^). Points: experimental data at given temperature, lines: Sips model.

As represented from Table 1, the *R*^2^ values indicated that Sips model has better fit with the experimental results then Langmuir and Freundlich models. Fig. 5 depicts the nonlinear functions of Sips model fitted to the experimental points obtained from the batch studies in different temperatures.

**Table 1:** Model parameters obtained from non-linear fitting the experimental equilibrium data with the isotherm models (adsorbent dosage 55 mg L^−1^, pH 7, contact time 60 min)

*T* (K)	*q*_exp_ (mg g^−1^)	Langmuir			Freundlich			Sips			
		*q*_m_ (mg g^−1^)	*K*_L_	*R*_L_^2^	*n*_F_	*K*_F_	*R*_F_^2^	*q*_m_ (mg g^−1^)	*K*_S_	*n*_S_	*R* ^2^
278	118	200.7	0.38	0.98	1.7	55.5	0.97	119.1	0.76	1.61	0.99
293	141	239.4	0.59	0.96	1.7	85.3	0.96	142.5	1.24	1.69	0.98
308	152	225.3	1.17	0.93	2.01	114	0.93	151.6	2.12	2.36	0.99
323	163	243.1	1.54	0.93	1.96	142	0.93	164.3	3.06	2.38	0.99

The Sips equation containing three parameters having the capability to apply for both the homogeneous and heterogeneous systems.^56^ The surface heterogeneity of adsorbent should be considered if the deviation of *n*_S_ values from 1 is observed.^55,57^ However, the Sips isotherm reach a constant level at high concentrations while a pattern of Freundlich model can be observed at low concentrations.^57^

As revealed from Table 1, the Pb^2+^ adsorption capacities of Fe_3_O_4_@DHAQ_GO nanocomposite were increased by the increasing of temperature assigning to decrease water viscosity along with the increasing of Pb^2+^ collisions between the sites of nanocomposite and Pb^2+^ ions. The maximum adsorption capacities (*q*_m_) obtained by Langmuir isotherm were overestimated (*e.g.* 243.1 in 323 K) while those of Sips model (*e.g.* 164.3 in 323 K) represents a good fit to the experimental data (also, see Fig. 5) which can be due to the heterogeneity characteristic considered in the Sips model.^58^ Increasing the deviations of *n*_S_ and *n*_F_ values from unity can be assigned to develop the nanocomposite surface heterogeneity over raising the temperature.^57^

#### 3.2.2. Kinetic studies

The sorption capacities (*q*_*t*_) of Fe_3_O_4_@DHAQ_GO exposed with Pb^2+^ initial concentrations 2.5, 5, and 10 mg L^−1^ were studied over corresponding times. The kinetic models; Lagergren-first-order (eqn (9)) and pseudo-second-order (eqn (10)) were applied for determining the appropriate function to describe the kinetic behavior of the batch systems.

9 *q*_*t*_ = *q*_e_(1 − exp(−*k*_1_t))

10
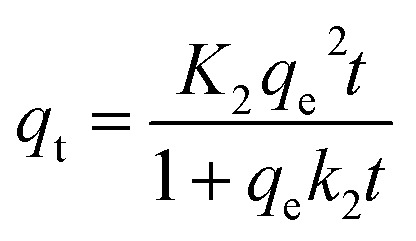
where, *q*_*t*_ and *q*_e_ are the sorption capacity (mg g^−1^) at time *t* and at the equilibrium time, respectively. *k*_1_ and *k*_2_ correspond to the pseudo-first-order and pseudo-second-order rate constants, respectively.^59,60^

**Fig. 6:**
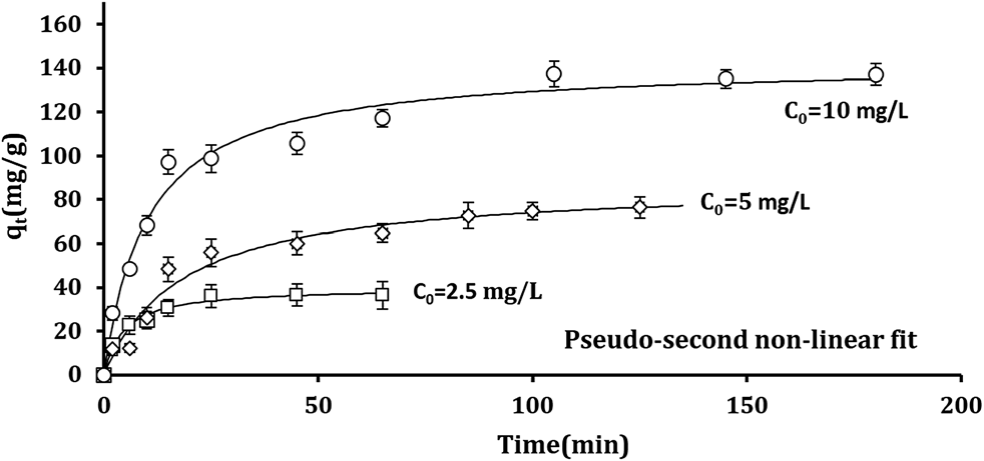
Nonlinear forms of pseudo-second kinetic model fitted on experimental points at different Pb^2+^ initial concentrations (adsorbent dosage 100 mg g^−1^; volume of solution 100 mL; pH 7; *T* = 298 K).

Fig. 6 illustrates fitting the non-linear forms of pseudo-second kinetic model to the experimental points. As shown, the equilibrium was took place sooner for the batch systems underwent lower Pb^2+^ initial concentrations.

Table S1† presents kinetic parameters of Pb^2+^ removal obtained by using the non-linear forms of pseudo-first and pseudo-second kinetic models (eqn (9) and (10)). As found in Table S1,† according to the *R*^2^ values, the pseudo-second model has better fit to the experimental points and *K*_2_ are increased by increasing the temperature, both are the evidences favor the chemisorption occurring.^61–63^

#### 3.2.3. Thermodynamic parameters

Changing in free energy (Δ*G*^0^), enthalpy (Δ*H*^0^), and entropy (Δ*S*^0^) can be determined by the following equations:

11 Δ*G*^0^ = −*RT* ln *K*_c_

12
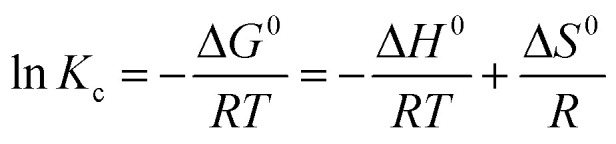
where, *R* is the gas constant (8.314 J mol^−1^ K^−1^), *K*_c_ (*q*_e_/*C*_e_) is equilibrium constant at different temperatures, and *T* is the absolute temperature (K). Eqn (11) calculates Δ*G*^0^ values assigning to the obtained temperature shown in Table 2.

**Table 2:** Thermodynamic parameters for the adsorption of Pb^2+^ onto the Fe_3_O_4_@DHAQ_GO nanocomposite (adsorbent dosage 55 mg L^−1^, contact time 60 min, pH 7)

*T*	*K*_L_ (L g^−1^)	*q*_m_ (mg g^−1^)	Δ*G*^0^ (kJ mol^−1^)	Δ*S*^0^ (J mol^−1^ K)	Δ*H*^0^ (kJ mol^−1^)
278	0.388	200.7	−13.79	135.97	24.07
293	0.598	239.4	−15.58	—	—
308	1.178	225.3	−18.12	—	—
323	1.546	243.1	−19.73	—	—

**Fig. 7:**
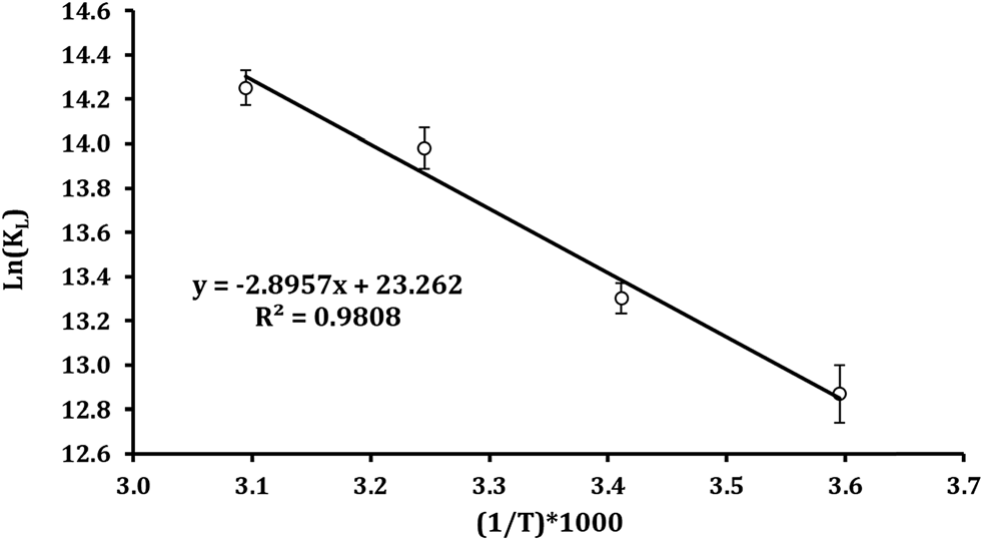
Effect of temperature on the adsorption of Pb^2+^ ions by Fe_3_O_4_@DHAQ_GO nanocomposite.

Enthalpy (Δ*H*^0^) and entropy (Δ*S*^0^) can be determined by plotting ln(*K*_c_) *versus* 1/*T* as revealed in Fig. 7. Furthermore, (Δ*H*^0^) and (Δ*S*^0^) can obtained be from the slope and intercept of linear form of eqn (12), respectively.^64–66^

Table 2 represents that Δ*G*^0^ has negative amounts assigning to different temperatures. So, it can be concluded that Pb^2+^ adsorption on Fe_3_O_4_@DHAQ_GO nanocomposite proceeds spontaneously.

Fan *et al.* (2013) found that obtained Δ*G*^0^ is ranged from −10.26 to −16.24 kJ mol^−1^ at 303–323 K.^67^ Also, Kumar *et al.* (2014) reported that the changes of free energy Δ*G*^0^ at 298 K are −6.46 kJ mol^−1^. As shown in Table 2, Δ*G*^0^ is −19.73 kJ mol^−1^ at 323 K having an appropriate agreement with the findings of Fan *et al.* (2013). Similar finding were reported by other researchers.^68–70^

As represented in Table 2, increasing the temperature tends to lower values assigned to Δ*G*^0^ confirming that the adsorption is more efficient at the higher temperatures.^71,72^ The enthalpy (Δ*H*^0^) value was 24.07 kJ mol^−1^ having the positive value of Δ*H*^0^ that indicates the endothermic nature of the adsorption. The entropy (Δ*S*^0^) was obtained with a positive value proving the increase of randomness during Pb^2+^ adsorption process.^73,74^

### 3.3. Selectivity study

Two independent studies were conducted to evaluate the selectivity properties of Fe_3_O_4_@DHAQ_GO nanocomposite for the separation of Pb^2+^ ions from aqueous ion matrices. The first one was capturing Pb^2+^ ions from four different aqueous solutions so that each solution contains Pb^2+^ and one other divalent metal ion. Consequently, four binary ion matrices were prepared, including Pb^2+^/Cu^2+^, Pb^2+^/Cd^2+^, Pb^2+^/Zn^2+^, and Pb^2+^/Co^2+^.

The second study was conducted for the assessment selective removal of Pb^2+^ in drinking water samples containing natural ion matrices. Certain amounts of Pb^2+^ ion were spiked into 30 different drinking water samples collected from various groundwater sources. Batch experiments were conducted based on the optimized values of variables pH, dosage, temperature, and the initial concentration.

#### 3.3.1. Selective removal of Pb^2+^ from binary ion matrices

The above mentioned aliquots containing binary ions were exposed to the functionalized (Fe_3_O_4_@DHAQ_GO) and pristine (GO@SiO_2_–Fe_3_O_4_) nanocomposite through independent batch experiments. Table 3 shows the results of distribution coefficient *K*_d_ (mL g^−1^), selectivity coefficient *k*, and the relative selectivity coefficient *k* obtained from eqn (2)–(4), respectively. As observed, the values of selectivity coefficient *k* is more than 19 for all binary ion comparisons. It means that Fe_3_O_4_@DHAQ_GO nanocomposite has a more notable preference for capturing Pb^2+^ ions compared with that of coexistence ions. For instance, Fe_3_O_4_@DHAQ_GO nanocomposite could capture Pb^2+^ ions 19.66 times more selectively than Cu^2+^ ions. Cai *et al.* reported a *k* value of 11.66 for Pb^2+^/Cu^2+^ binary ions. Furthermore, Msaadi *et al.* and Zhu *et al.* reported similar findings for Pb^2+^ ions removal using ion-imprinted nanocomposites.^75,76^

**Table 3:** Selectivity parameters of Pb^2+^ comparative loading by Fe_3_O_4_@DHAQ_GO and Fe_3_O_4_@SiO_2_–GO sorbents at pH 7, and *T* = 298 K (acetic acid/sodium acetate buffer)

Metal ion	Fe_3_O_4_@DHAQ_GO			GO@Fe_3_O_4_			*K*
	*q*_e_ (mg g^−1^)	*k*_d_ (L g^−1^)	*K*_Fe_3_O_4_@DHAQ_GO_	*q*_e_ (mg g^−1^)	*k*_d_ (L g^−1^)	*k*_GO@SiO_2__–_Fe_3_O_4__	
Pb^2+^	132	41.88	19.37	47	8.14	0.99	19.66
Cu^2+^	15	2.16		50	8.26		
Pb^2+^	133	36.81	31.89	37	5.74	1.34	23.72
Ni^2+^	9	1.15		30	4.27		
Pb^2+^	123	34.73	19.41	24	3.67	0.71	27.20
Co^2+^	12	1.79		32	5.15		
Pb^2+^	119	32.38	15.09	21	3.21	0.58	26.09
Cd^2+^	15	2.15		35	5.54		

#### 3.3.2. Selective removal of Pb^2+^ from drinking water samples

Table S2† shows a set of multiple regression models ranked according the Akaike's Information Criterion (AIC). Table 4 represents the coefficients of the model obtained rank 1 in Table S2.† As observed, cations formed the drinking water matrices (Na^+^, K^+^, Ca^2+^, Mg^2+^) obtained negative values confirming their competition with Pb^2+^ ion to occupy the active sites of Fe_3_O_4_@DHAQ_GO nanocomposite. The large value assigned to the intercept (105.47) ensured notable preference of Fe_3_O_4_@DHAQ_GO nanocomposite for the separation of Pb^2+^ ion from drinking water.

**Table 4:** Ranking list of linear multiple regression models applied to describe the effect of main natural water ions on mercury removal efficiency by Akaike's Information Criterion (AIC)

Model elements^a^	Coefficient	Standard error	*T* value	Pr(>|*t*|)	*P*-value
Intercept	105.47	5.23	20.15	0.0001	0.001
NO_3_^−^	0.70 (a)	0.43	1.62	0.120	0.1
SO_4_^2−^	0.17 (b)	0.05	2.8	0.009	0.001
Cl^−^	0.31 (c)	0.08	3.66	0.001	0.001
HCO_3_^−^	0.15 (d)	0.04	3.51	0.002	0.001
Na^+^	−0.29 (e)	0.14	−1.95	0.064	0.05
K^+^	−0.47 (f)	−0.27	−1.75	0.093	0.05
Mg^2+^	−1.15 (g)	−0.29	−3.89	0.0008	0.001
Ca^2+^	−0.73 (h)	−0.15	−4.69	0.0001	0.001

a Multiple *R*^2^: 0.81, adjusted *R*^2^: 0.73.

### 3.4. Desorption and regeneration

**Fig. 8:**
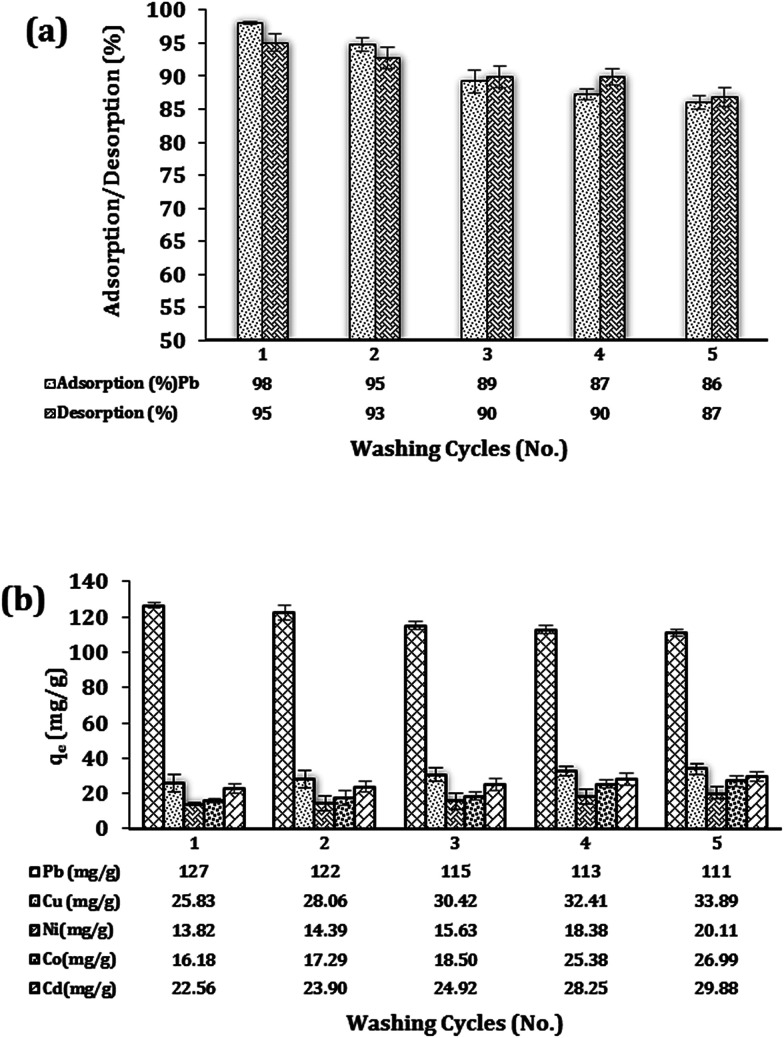
Reusability studies; repeated adsorption/desorption of Pb^2+^ by Fe_3_O_4_@DHAQ_GO nanocomposite (a). The consecutive adsorption capacities (mg g^−1^) of Fe_3_O_4_@DHAQ_GO nanocomposite for Pb(ii), Cu(ii), Ni(ii), Co(ii), and Cd(ii) ions during the five adsorption/desorption cycles (b). *C*_0_ ≃ 50 mg L^−1^, adsorbent dosage = 0.4 g L^−1^, pH 7, contact time 30 min, desorption agent: EDTA-2Na 0.01 N.

Fig. 8(a) depicts the repeated adsorption/desorption of Pb^2+^ ions using batch experiments exposed with Fe_3_O_4_@DHAQ_GO nanocomposite in single ion aqueous solution. As shown, after 5 consecutive regeneration steps, the nanocomposite could remove 86 percent of Pb^2+^ ions so that only 12 percent of removal loss was observed.

Fig. 8(b) shows the results of Fe_3_O_4_@DHAQ_GO regeneration study in an aqueous ion matrix consisting of five divalent metals. This experiment aims to investigate the presence of four coexistence ions (Cu^2+^, Ni^2+^, Co^2+^, Cd^2+^) in the case of their effect on lead removal and to assess the capability of the nanocomposite for the retaining of lead adsorption capacity after several washing steps in the presence of other cations. As observed, the removal capacities of Pb^2+^ ion was remained more than 111 mg g^−1^ over five regeneration steps using the desorption agent EDTA-2NA 0.01 N. Furthermore, increasing the sorption capacity assigned to the four coexistence ions were almost negligible confirming the notable stability of the nanocomposite structure over several regeneration experiments.

The stability of the Pb^2+^ and Fe_3_O_4_@DHAQ_GO complex was confirmed *via* the adsorption/desorption experiments. The conventional methods for evaluating the regeneration and reusability of adsorbents are according to the consecutive adsorption/desorption steps in batch volumes containing deionized water solution inoculated with the target pollutant. Consequently, the effects of coexistence ions are neglected, especially when the reusability of adsorbents having selectivity properties is considered.^77^

Here, we put forward a facile approach to investigate the reusability of Fe_3_O_4_@DHAQ_GO in aqueous ion matrices containing different competitor divalent cations (Fig. 8). Yu *et al.* reported applying EDTA-2Na 0.015 N as washing agent over three cycles regeneration steps. Results showed the notable interference of Cd^2+^ (ref. 78) while, in our work, the minimum interfering of the coexistence cations was observed.

## 4. Conclusions

In this work, a novel hydrophilic nanocomposite based on GO was synthesized comprising an anthraquinone derivative having selective removal capability for lead. Fe_3_O_4_ nanoparticles was used as a magnetic agent to facilitate the separation of nanocomposite from aqueous solution. Also, GO was used as a dispersible platform to obtain the hydrophilic property for the nanocomposite and preparing enough surface area to proceed the adsorptive mechanisms. The morphology and structure of the obtained adsorbent was characterized by UV-Vis, FT-IR, SEM, XRD, and TGA. The synthesis rout was simple and DHAQ was an environmental friendly compound without toxic effect. The selectivity characteristics of the nanocomposite was evaluated through two different methods including controlled ion matrices and the natural ion matrices obtained from drinking water samples. Furthermore, the regeneration and reusability studies were conducted in the presence of coexistence ions. It seems that Fe_3_O_4_@DHAQ_GO nanocomposite can be a promising selective removal agent for the removal of lead from polluted waters and industrial discharges.

## Conflicts of interest

There are no conflicts to declare.

## Supplementary Material

RA-008-C7RA13603J-s001
