# Supplementary material for: Selective removal of lead ions from aqueous solutions using 1,8-dihydroxyanthraquinone (DHAQ) functionalized graphene oxide; isotherm, kinetic and thermodynamic studies
Source: RSC Adv. 2018 Feb 2;8(11):5685–94. doi: 10.1039/c7ra13603j (PMC9078188; doi:10.1039/c7ra13603j)
Supplement: RA-008-C7RA13603J-s001 [file RA-008-C7RA13603J-s001.pdf]

Electronic Supplementary Material (ESI) for RSC Advances.
This journal is © The Royal Society of Chemistry 2018

Selective removal of lead ions from aqueous solutions using 1, 8-dihydroxyanthraquinon (DHAQ) functionalized graphene oxide; isotherm, kinetic and thermodynamic studies

Mohammad Khazaei

Department of Environmental Health Engineering, School of Public Health and Research Center for Health Sciences, Hamadan University of Medical Sciences, Hamadan, Iran

Department of Environmental Health Engineering, School of Public Health, Tehran University of Medical Sciences, Tehran, Iran.

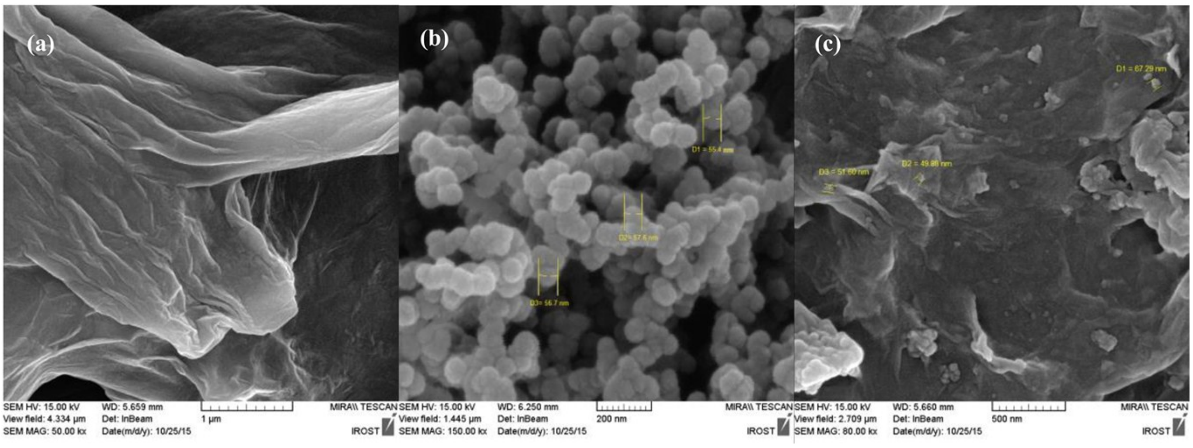

Figure S1. SEM images of GO (a), Fe_3O_4 nanoparticles (b), and Fe_3O_4@DHAQ_GO

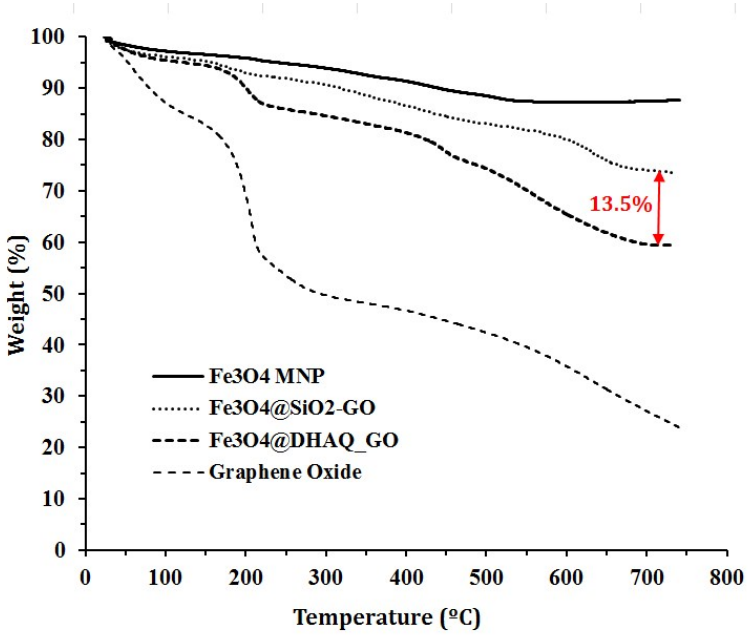

Figure S2. The TGA curves of Fe_3O_4 magnetic nanoparticles, Fe_3O_4@SiO_2-GO, Fe_3O_4@DHAQ_GO, and graphene oxide.

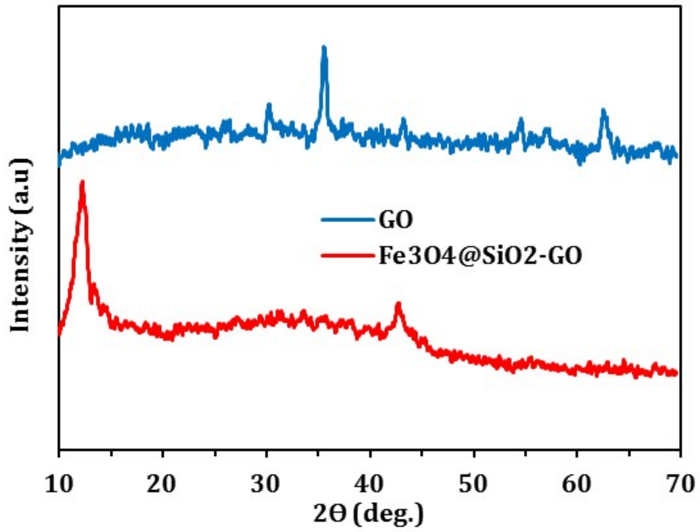

Figure S3. XRD patterns of GO (a) and Fe_3O_4@SiO_2-GO (b).

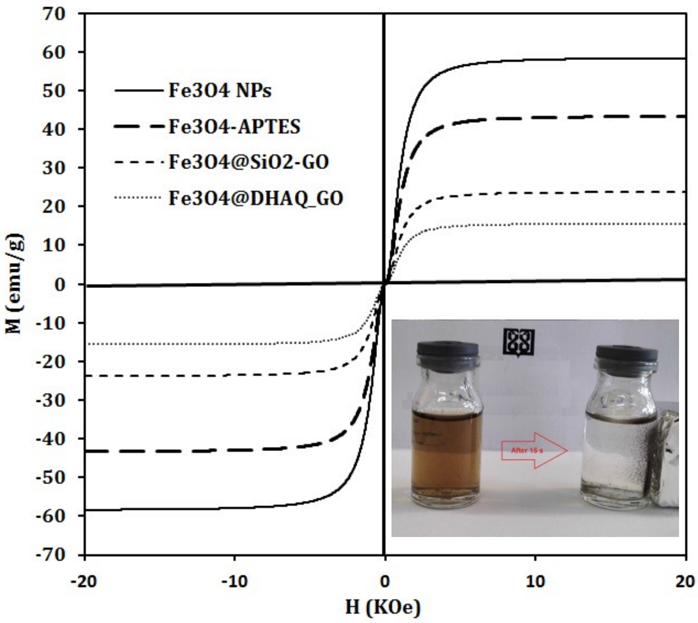

Figure S4. Hysteresis curves assigned to the Fe_3O_4 NPs (a), Fe_3O_4-APTES (b), GO@SiO_2-Fe_3O_4 (c), and Fe_3O_4@DHAQ_GO nanocomposite (d).

Table S1. The kinetic models for the adsorption of Pb^{2+} onto the Fe_3O_4@DHAQ_GO nanocomposite (adsorbent dosage 55 mg L^{-1}, pH 7).

T (K)	C_0 (mg/L)	Pseudo-First Order			Pseudo-Second Order		
		q_e	K_1	R^2	q_e	K_2	R^2
	2.5	35.9	0.153	0.972	39.9	5.50	0.988
**278**	5	72.8	0.052	0.968	87.6	0.63	0.969
	10	127.5	0.077	0.956	142.7	0.69	0.981
	2.5	38.9	0.114	0.829	42.4	4.43	0.891
**293**	5	82.3	0.077	0.951	92.9	1.08	0.967
	10	134.5	0.085	0.829	149.7	0.76	0.903
	2.5	39.7	0.323	0.928	43.2	11.45	0.961
**308**	5	85.1	0.092	0.961	94.4	1.34	0.978
	10	155.0	0.103	0.944	169.5	0.86	0.974
	2.5	41.2	0.695	0.948	44.1	22.04	0.965
**323**	5	88.3	0.116	0.970	95.3	1.91	0.972
	10	161.3	0.157	0.973	174.3	1.30	0.987

Table S2. Ranking list of linear multiple regression models applied to describe the effect of main natural water ions on Pb^{2+} removal by Akaike's Information Criterion (AIC).

Model	DF[1]	SS[2]	RSS[3]	AIC	Rank
Y^*=f(x^{**})	-	-	290.69	86.131	1
y=f(x)+f(F^-)	1	9.741	280.95	87.109	2
y=f(x)-f(NO_3^-)	1	36.322	327.01	87.664	3
y=f(x)-f(K^+)	1	42.758	333.44	88.248	4
y=f(x)-f(Na^+)	1	52.605	343.29	89.121	5
y=f(x)-f(SO_4^{2-})	1	113.585	404.27	94.027	6
y=f(x)-f(HCO_3^-)	1	171.305	461.99	98.03	7
y=f(x)-f(Cl^-)	1	185.74	476.43	98.953	8
y=f(x)-f(Mg^{2+})	1	210.069	500.76	100.448	9
y=f(x)-f(Ca^{2+})	1	304.764	595.45	105.644	10

P-value $_{\text{(Shapiro-Wilk normality test for Pb(II) Removal (-log(Ct/C0))}}$ = ***0.094***
1.Degree of Freedom
2. Sum of Squares
3. Residual Sum of Squares
*y: Pb^{2+} Removal (-log(C_t/C_0))
**x: a×NO_3^- + b×K^+ + c×Na^+ + d×SO_4^{2-} + e×HCO_3^- + f×Cl^- + g×Mg^{2+} + h×Ca^{2+}
Model coefficients: *a, b, c..., h.*
Ions concentration unit: mg/L
